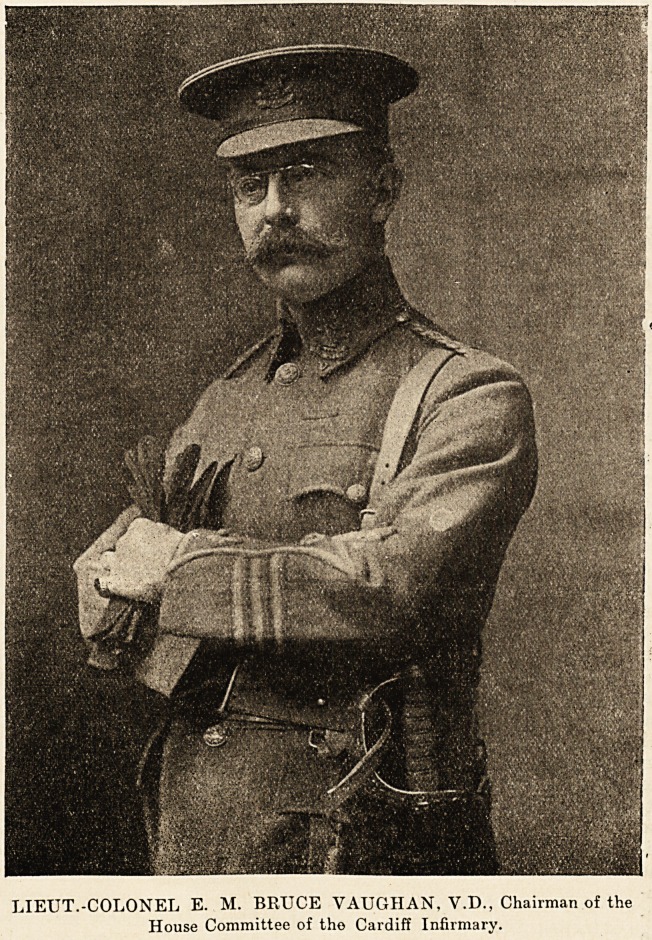# Eminent Chairmen Series
*Previous articles in this series appeared in The Hospital of Oct. 1, Nov. 1, Dec. 10, Jan. 14, Feb. 11, March 11, April 22, and May 20.


**Published:** 1911-07-29

**Authors:** 


### SPECIAL INSTITUTIONAL ARTICLE.

### EMINENT CHAIRMEN SERIES.*

### IX.—LIEUT.-COLONEL E. M. BRUCE VAUGHAN, V.D., Chairman of the House Committee of the Cardiff Infirmary.

For nearly ten years the name of Colonel Bruce Vaughan has been so closely identified with the Cardiff Infirmary that they have come to be regarded as interchangeable terms, and the mention of either inevitably suggests the other, and always to the credit of both. It is interesting to discover what has first attracted a great hospital worker to the object of his solicitude and constant care, and there would appear to be little doubt that in this instance a friendship with members of the infirmary staff had led to occasional visits to the hospital and a personal knowledge of the value of its work. By slow gradation, but very thoroughly, this knowledge indicated in what direction the natural kindness of a true heart might find its best and most useful expression, and it is characteristic of Colonel Vaughan that it was in days when the institution was unpopular — in days of despondency and depression—that he first took a conspicuous interest in the affairs of the Cardiff Infirmary. In or about the year 1898 the Cardiff Infirmary was made the object of a ceaseless attack, and powerful public influences were brought to bear in discrediting a useful and honourable, if, at that time, somewhat unprogressive, institution. Colonel Bruce Vaughan happened to know, by reason of his frequent visits to the Infirmary, that the allegations brought against it were without foundation, and, at a time when it was not fashionable to contribute to its funds, he sent along a substantial donation as a mark of his personal belief in its integrity.

LIEUT.-COLONEL E. M. BRUCE VAUGHAN, V.D., Chairman of the House Committee of the Cardiff Infirmary.

#### A Retrospect.

Perhaps a brief retrospect of the history of the Infirmary up to this crisis may prove of interest and serve to present in a clearer light the value of Colonel Bruce Vaughan's subsequent services to the institution. In succession to a modest dispensary the Cardiff Infirmary was established in the year 1837, upon the initiative of the then Marquess of Bute—the grandfather of the present peer—who provided a site for the building and a handsome sum towards its maintenance, whilst the entire cost of its erection was borne by the late Mr. Daniel Jones, of Beaupre. This building was capable of accommodating forty beds, and for many years proved of great usefulness to the community, when the phenomenal growth of the population of the district called imperatively for increased hospital accommodation, and it was found necessary to erect a new and larger building on a different site, where extension was possible. The new building, providing accommodation for 108 patients, was

* Previous articles in this series appeared in The Hospital of Oct. 1, Nov. 1, Dec. 10, Jan. 14, Feb. 11, March 11, April 22, and May 20.

placed at the disposal of the public in 1884, and the old building then became the home of the University College. An extension was added to the new building in 1896, providing accommodation for 64 additional beds, bringing the total capacity of the hospital to 172 beds. There followed years of marked increase in the population of Cardiff and the wide district served by the Cardiff Infirmary. It is computed that within the geographical limits of this area there are quite half a million of people, whilst the hospital accommodation of South Wales is notorious in its inadequacy. Sir Henry Burdett, counting all the beds provided at Cardiff, Swansea, and Newport, as well as in the various cottage hospitals in the district, computes there is a deficiency in reasonable requirements of 2,000 beds.

Although the demand made upon the Infirmary was daily increasing little was being done to meet it. The support of the institution was lukewarm and indifferent, and by the year 1900, when increased hospital accommodation was more than ever required, no fewer than 50 of the available beds were closed down, and even then the annual expenditure of the institution exceeded its income. It was during this period that Colonel Bruce Vaughan became a member of the Committee, but no one could have anticipated from the quiet and unostentatious manner in which he occasionally interpolated a few remarks in debate that he would become an active force in hospital reorganisation. His unwearied pursuit of information might have warned one, but it is at any rate certain that the suggestions he advanced were generally regarded as not coming within the sphere of practical politics. Colonel Bruce Vaughan was not, however, idle. He was evolving a programme so comprehensive and so far seeing that, had he announced it in its entirety, the Committee of the Infirmary would have regarded him as a dangerous enthusiast. It was a programme first of all to render the existing buildings efficient, then of extension, then to promote an increase of income coupled with a greater efficiency of administration, and ultimately extending to secure an improved status for the hospital in association with the foundation and development of a medical school in connection with the local university.

### The Heralds of Brighter Days.

It is only fair to mention that three circumstances during this period contributed largely to Colonel Bruce Vaughan's ultimate success. In the year 1901 Mr. Thomas Andrews, a generous and public-spirited Mayor of Cardiff, announced at the Mayoral banquet his intention to clear off during his year of office the debt which was crippling the work of the Infirmary. Mr. Andrews' initiative was warmly taken up by the *Western Mail*, which opened a shilling fund in its popular columns, and, well within the stipulated time, a sum sufficient to pay the overdraft at the bank was contributed — namely, £12,568 13s. 11d. This magnificent effort had also the valuable effect that public attention and sympathy were directed to an especial degree and in an attractive manner to an institution now becoming recognised as in every way worthy of public support. As indicating this awakening interest in the Infirmary, Lady Aberdare and other ladies of the county were greatly distressed when they realised that so many poor women were drifting towards permanent invalidity (if, happily, their lives were spared) owing to the delay in obtaining admission to the Infirmary. Through the sustained efforts of these charitable ladies, one of the empty wards was opened for the reception of gynæcological patients, and all the beds in this ward have since been maintained—some by permanent endowment and the others by yearly contributions—in many instances a bed being supported by the contributions from a defined district. The third helpful circumstance was the splendid gift of a much needed operating theatre by the late Mr. Thomas Webb, a gentleman largely interested in the development of the colliery industry and who was ever mindful of the source of his wealth and careful to consider the claims of the locality upon his generosity. Colonel Bruce Vaughan was an active member of the Building Committee appointed in connection with the erection of Mr. Webb's operating theatre, and in this respect his professional experience (Colonel Vaughan is an architect) proved of great value to the institution.

### Building Additions and Improvements.

The operating theatre completed, Colonel Bruce Vaughan devoted the next few years to building improvements and to extensions which did not at this stage contemplate the addition of beds. He fully recognised that more beds were urgently needed, but he felt strongly that it was first of all necessary to render the existing buildings efficient. This necessity was obvious in every direction, and in almost every department the skill and devotion of the honorary medical staff were prejudiced by an absence or an inadequacy of the requirements of modern surgical practice. Colonel Vaughan initiated his scheme of reform in an entirely modest and practical manner. He made it clear that a modern and complete installation of steam and gas cooking and other appliances for the hospital kitchen were highly desirable, as well as the provision of new, well-ventilated pantries. The Committee quite agreed, but absolutely refused to sanction the expenditure. Colonel Vaughan was disheartened, and there followed a simple circumstance which completely altered his attitude towards the task he had set himself, and immensely added to his responsibility. After the meeting, the Chairman of the Board of Management, Major-General Lee, expressed his sympathy in the disappointment, and offered the suggestion that Colonel Vaughan might himself collect the money required and he (General Lee) would contribute £50 towards it. After consideration Colonel Vaughan acted upon this suggestion, and he has adopted it in every work (and their name is legion) that he has subsequently undertaken for the Infirmary.

It is a matter for speculation whether he would have succeeded in his plans to such a degree had he continued to be merely an ordinary member of the Committee, for in 1903, yielding to the solicitation of General Lee and the unanimous request of his colleagues, he became the Chairman of the House Committee, which is in practice the chief executive committee of the institution. He has continued in this office ever since, greatly to the advantage of the hospital, but never failing to acknowledge the great help he has at all times received from the unfailing consideration and sympathy of the gallant Chairman of the Board of Management, Major-General Lee.

### How the Expenses were Defrayed.

The money for the pantries and kitchen equipment was soon raised, and repeated applications to the Committee for assistance to carry out further improvements proving entirely futile, Colonel Vaughan determined to provide for the requirements independently of the Committee, merely asking for their assent to his proposition that the requirements were highly desirable. This assent was readily given, though perhaps rather perfunctorily, as many of the members regarded the realisation of the schemes as visionary. Then Colonel Vaughan, at great pains and with infinite care, prepared a list, giving brief details of the additions and improvements required, stating that these arose as much from the age of the hospital building as from the necessity for providing those adjuncts which form an integral part of every modern hospital, and claiming that the proposals had received the most careful consideration of both Medical and Lay Committees, and were regarded by them as absolutely essential to the efficiency of the hospital. Opposite each item was stated the amount which it was estimated to cost, and a hope was expressed that friends of the Infirmary would identify themselves with one or other of these much needed improvements. The hope was not expressed in vain, and in rapid succession the various items of the programme were subscribed for, the present Marquess of Bute leading the way by defraying the cost of electrically lighting the entire institution, whilst the late Mr. Thomas Webb again came to the rescue by providing a new and well adapted casualty department. Viscount Tredegar, with characteristic generosity, made himself responsible for the erection of verandahs to all the wards, which have since proved a veritable blessing. The late Mrs. W. H. Martin bore the expense of a pavilion for *x*-rays and the electrical treatment of patients. The entire institution was equipped with a hot-water and heating plant, the cost of which was largely defrayed by a legacy bequeathed to the institution by the late Mr. Alfred Brownett, a Cardiff working man, and on the same lines many smaller, but no less useful, works were carried out, greatly to the comfort of the patients and to the efficiency of their treatment.

### Every Available Bed Placed in Commission.

Colonel Vaughan, having thus whetted his appetite for constructive work, paused to ensure that the remaining ward space of the existing buildings should no longer lie idle. This and the conversion of the day rooms into bed accommodation increased the number of beds to 196, and, inspired by Colonel Vaughan's cheery optimism and the increasing success of his efforts, the Committee ultimately placed every possible bed at the disposal of the expectant patients.

The growing popularity of the hospital and the extension of industrial pursuits had increased the waiting list to over three hundred patients, and it was apparent that a new wing was essential to keep pace with the needs of the district, but with characteristic prudence Colonel Vaughan was determined that, before this large expenditure should be incurred, the improvement of the out-patient department should be dealt with, an improvement in its own way no less urgent than the provision of new beds. The out-patient department was the only portion of the original building which had not been altered to meet the very different requirements which it had now to fulfil, compared with those obtaining when it was erected. The accommodation was insufficient, the building badly lighted and ill-ventilated, and though at the time it was built it was considered quite satisfactory, it was now recognised as being utterly unfitted for the modern treatment of disease. The rooms were so inconvenient and necessarily used for so many different classes of cases that the long hours, during which many patients were obliged to wait their turn to see the out-patient staff, seriously affected their condition, and it became a subject of public reproach. These facts were made clear by Colonel Vaughan's eloquent and incisive appeal when a "Glamorganshire Owner of Property" (than whom the Infirmary possesses no truer or more generous friend) offered £1,000 provided the balance required was subscribed within a stipulated time. The "Glamorganshire Owner of Property" had also an eye to the future, for it was stated that the money was given, not only for the purpose of remodelling the out-patient department, but also of thereby removing the last and most serious obstacle to providing the increased accommodation necessary for treating the ever-increasing number of patients waiting admission. The response to the appeal was remarkable, and within a few months £8,000 was subscribed, and in due time the old out-patient department was replaced by a building which is generally recognised as one of the most highly convenient and best adapted for its purposes to be found in the provinces. Incidentally the upper story of the renovated building provides accommodation for additional nurses.

In the appeal for the out-patient department and, indeed, in all his subsequent appeals, Colonel Vaughan was greatly assisted by the benevolence and influence of Lord Merthyr of Senghenydd (then Sir William Thomas Lewis), and the foundation-stone of the out-patient building was laid by Sir William on July 30, 1907.

### The New Wing.

The remodelling of the out-patient department completed and out of the way, the question of the new wing was vigorously faced, and owing to the munificence of the late Mr. John Cory, who promised £5,000 towards the extension, an inspiring start was made, and within an incredibly short space of time, the sum required—namely, £30,000—was completed by another munificent gift of £5,000, this time at the generous hands of Mr. H. Woolcott Thompson.

The new wing, the structure of which is now, happily, completed and practically ready for occupation, will in itself form an abiding monument to Colonel Vaughan's skill and devotion. It is a wonderful building, or rather series of buildings, and provides many other requirements in addition to the most up-to-date ward accommodation for 82 beds, fitted with verandahs and fire-escape equipment. For instance, there are a complete isolation block at the top of the building, cut off from the rest of the institution and approached from the outside by a separate entrance, staircase and patients' lift; a new corridor connecting the first floors of all the wards in the old building; lifts for patients, coal and food, to serve the entire institution; a room for delirious patients; a library and lecture-room for nurses; doctors' and nurses' dining-rooms; sleeping accommodation for 34 additional nurses and 22 additional servants; disinfector house; store rooms and other items; as well as the "Lady Aberdare" operating theatre, which was described by Sir Francis Champneys recently upon the occasion of its opening as "containing many new features, which placed it a step in front of probably all existing theatres." There is also a magnificent pathological block, three stories high, and with basement equipped with freezing apparatus, possessing the most up-to-date arrangements for carrying out this important branch of hospital work, not only to meet ordinary hospital requirements, but also the fullest demands of the developing Medical School of the Cardiff University College. The provision of this pathological block assisted in inducing the Treasury to make an additional grant of £15,000 a year to the University of Wales, of which sum £1,500 is allocated for the foundation of a Cardiff Medical School.

### Increasing the Income.

Before a stone of the new wing was laid, Colonel Vaughan had already entered on the most arduous campaign of all his hospital career—the acquisition of a permanent income to maintain the proposed building. It was estimated that £7,000 additional annual income was required to maintain, free of debt, the entire hospital when completed in every detail. The income requisite to maintain the old building was deficient by £2,000 a year, and the overdraft at the bank had mounted up to £22,000, but Colonel Vaughan's policy was to leave the overdraft alone for the moment, as its removal would not in itself prove a permanent cure. This had been the experience in 1901 when Mr. Thomas Andrews' efforts had removed the overdraft then existing, leaving the public under the misapprehension that they had done everything, and freed the Infirmary for all time. The Colonel was determined upon effecting a radical cure—namely, to remove the cause of the overdraft, the disparity between the income and expenditure. Efforts were initiated to practise economy in every direction, but salvation lay chiefly in increasing the income. He appealed to every section of the community—to the wealthy, for money to endow beds, and to the less wealthy, and especially to the working men and colliers of the district, for annual subscriptions and systematic weekly collections. He appealed for a capital sum of £175,000, or its equivalent in annual subscriptions, and by the time that Mrs. John Nixon, one of the earliest and most generous to respond to this appeal, laid the foundation-stone of the new wing, £3,500 additional annual income—or half the requisite amount—was already assured, either from the investment of additional capital contributed, or from increased annual subscriptions or collections. Mrs. John Nixon gave £10,500, and other notable contributors were Mr. William James Thomas, J.P., of Ynyshir, £10,500, and "A Glamorgan Coal Owner," £10,000.

"Cardiff City Memorial to King Edward VII."

This is the same appeal that has become sanctified by the death of King Edward, and the outcome of which is to be regarded as a memorial to the beloved Sovereign who had become, to use Colonel Vaughan's own expression, the "Grand Almoner of the Nation." The only difference arises from the fact that the primary object of the Cardiff City Memorial to King Edward VII. is to liquidate the debt upon the Infirmary, as it is felt that the approbation of King George would not rest upon a form of Memorial sullied by debt; but the remainder of the sum that is asked for will be devoted to the permanent capital of the institution, with the view of providing an additional income that will be free from the disadvantages of uncertain and spasmodic gifts, mainly dependent upon the fluctuations of commercial enterprise.

Cardiff is ambitious that, by the accomplishment of these most desirable ends, his Majesty King George will give his consent to re-name the hospital "King Edward VII.'s Hospital, Cardiff," and it is singularly appropriate that the first response to the Memorial to King Edward came from the Marquess of Bute, who is contributing the munificent sum of 6,000 guineas. It was in Cardiff Castle, in the month of August 1834, that the nucleus of a fund for the erection of a public infirmary was set aside for this purpose, the money representing the balance remaining after defraying the expenses of the Gwent and Dyfed Royal Eisteddfod, which was held in the Castle at Cardiff under the patronage of her late Majesty Queen Victoria, then the Princess Victoria. Her Majesty Queen Victoria's accession to the throne synchronised with the opening of the institution, and it is the earnest hope of the Governors of the Infirmary that his Majesty King George's gracious favour will preserve to their hospital this royal tradition.

The wise and imaginative foresight of one man, combined with his unfailing and unselfish devotion, has rendered possible the realisation of this hope, and that man is the subject of this all too inadequate sketch, which has only been able to touch upon a few of his many notable services to the Cardiff Infirmary.

## Figures and Tables

**Figure f1:**